# *vacA* Genotype Status of *Helicobacter pylori* Isolated from Foods with Animal Origin

**DOI:** 10.1155/2016/8701067

**Published:** 2016-03-07

**Authors:** Elnaz Saeidi, Amirhossein Sheikhshahrokh

**Affiliations:** Biotechnology Research Center, Islamic Azad University, Shahrekord Branch, Shahrekord 8815774471, Iran

## Abstract

According to controversial theories and results of studies, foods with animal origins play an important role in the transmission of* H. pylori* to human. The aim of this study was to determine the distribution of* vacA* genotypes of* H. pylori,* isolated from milk and meat samples of cow, sheep, goat, camel, and buffalo. Eight hundred and twenty raw milk and meat samples were collected from various parts of Iran. Samples were cultured and those found positive for* H. pylori* were analyzed for the presence of various genotypes of* vacA* gene. Out of 420 milk and 400 meat samples, 92 (21.90%) and 105 (26.25%) were positive for* H. pylori*, respectively. The most commonly detected genotypes in the* vacA* gene were* s1a* (86.80%),* m1a* (79.18%),* s1b* (69.54%), and* m1b* (63.45%) and detected combined genotypes were mostly* m1as1a* (68.52%),* m1as1b* (60.40%),* m1bs1b* (55.83%), and* m1bs1a* (53.29%). High presence of bacteria in the milk and meat samples of sheep represents that sheep may be the natural host of* H. pylori*. High presence of* H. pylori* strains in milk and meat samples similar to* vacA* genotypes in human being suggests that milk and meat samples could be the sources of bacteria for human.

## 1. Introduction

Although* Helicobacter pylori* (*H. pylori*) has been accepted as a major cause of gastrointestinal disorders and especially gastric adenocarcinoma, type B gastritis, mucosa associated lymphoid tissue lymphoma, and peptic ulcer disease, its route of transmission, sources, and also the role of foods are still unknown.* H. pylori* is a Gram negative, coccoid flagellated bacterium with 2 to 4 *μ*m in length and 0.5 to 1 *μ*m in width, which is the first formally recognized bacterial carcinogen and one of the most successful human pathogens [1–5]. High prevalence of* H. pylori* in the stomach of domestic animals, milk, meat, and gastric biopsies suggests that food with animal origin, and also domestic animals, may be its reservoirs [1–5]. Higher prevalence of* H. pylori* in meat eaters than vegetarians which was achieved in the previous investigation supports the significant role of foods with animal origins in the transmission of bacteria to humans [6]. Appropriate condition of meat and milk including acidic PH, nutritional values, salt concentration, and also high amount of activated water (AW) facilitate the growth and survival of* H. pylori* and provide adequate setting for transmission of* H. pylori* to human [7]. High prevalence of antibodies against* H. pylori* in the serum samples taken from veterinarians, butchers, and staffs of the slaughterhouses and milking rooms can support the zoonoses aspects of this bacterium [1, 8].

To evaluate the pathogenicity of* H. pylori*, apprising virulence factors is requisite. The most commonly identified virulence factor among* H. pylori* strains is vacuolating cytotoxin (*vacA*) [9, 10].* VacA* belongs to the group of genes with mutable genotypes associated with damage to gastric epithelial cells. This gene exists in practically all strains of* H*.* pylori*. This gene is polymorphic and comprises variable signal regions (type* s1* or type* s2*) and midregions (type* m1* or type* m2*) [9, 10]. The s-region is classified into* s1* and* s2* and the m-region is categorized as* m1* and* m2*. The* s*1 type is further subtyped into* s1a*,* s1b,* and* s1c* and the subcategories of* m1* are* m1a* and* m1b,* respectively. Higher cytotoxicity and acuity have been done by this mosaic pattern [11, 12]. Genotyping using* vacA* alleles is considered as one of the best methods to study the associations of* H*.* pylori* strains in various samples.

Industrialized information designated the fact that closely 50% of the world population and also 60–90% of Iranian people are infected with virulent strains of* H. pylori* [4, 13]. According to the high prevalence of* H. pylori* in Iran and other parts of the world, and also with respect to the indistinct situation of* H. pylori* in foods with animal origins, the present investigation was carried out in order to study the* vacA* genotype status of* H. pylori* isolated from Iranian raw milk and meat samples.

## 2. Materials and Methods

### 2.1. Sample Collection

In all, 420 raw milk samples were collected: cow (*n* = 120), sheep (*n* = 120), goat (*n* = 80), buffalo (*n* = 50), and camel (*n* = 50) raw milk samples were collected from farm bulk tanks and milk collection centers from several geographic regions of Iran, from March 2013 to March 2014. Cow and buffalo milk samples were collected throughout this time period. Because the lactating periods of ewes and goats in Iran are seasonal (from March through May and September to November of the subsequent year), goat and sheep milk samples were only available through these months within the fore-mentioned time frame. At each site, sampling of milk was performed according to the International Dairy Federation guidelines (IDF 1995). Samples (100 mL, in sterile glass containers) were transported to the laboratory at ca. 4°C within a maximum of 6–12 h after sampling. For raw meat samples, 100 cow, 100 sheep, 100 goat, 50 buffalo, and 50 camel meat samples were purchased from butchers of various parts of Iran. All samples were kept under refrigeration in plastic bags; information about dates of production and assigned shelf lives was not presented. Meat samples were collected over a period of eight months from August 2013 to February 2014, and they were analyzed on the day of acquisition. Samples were transported under refrigeration (4–6°C) in thermal boxes containing ice packs and were tested immediately after collection.

### 2.2. Isolation of* Helicobacter pylori*

Twenty-five mL of each homogenized sample was added to 225 mL of Wilkins Chalgren anaerobe broth (Oxoid, UK) supplemented with 5% of horse serum (Sigma, St. Louis, MO, USA) and colistin methanesulfonate (30 mg/L), cycloheximide (100 mg/L), nalidixic acid (30 mg/L), trimethoprim (30 mg/L), and vancomycin (10 mg/L) (Sigma, St. Louis, MO, USA) and colistin methanesulfonate (30 mg/L), cycloheximide (100 mg/L), nalidixic acid (30 mg/L), trimethoprim (30 mg/L), and vancomycin (10 mg/L) (Sigma, St. Louis, MO, USA) and incubated for 7 days at 37°C with shaking under microaerophilic condition. Then, 0.1 mL of the enrichment selective broth was plated onto Wilkins Chalgren anaerobe agar (Oxoid, UK) supplemented with 5% of defibrinated horse blood and 30 mg/L colistin methanesulfonate, 100 mg/L cycloheximide, 30 mg/L nalidixic acid, 30 mg/L trimethoprim, and 10 mg/L vancomycin (Sigma, St. Louis, MO, USA) and incubated for 7 days at 37°C under microaerophilic condition. For comparison, a reference strain of* H. pylori* (ATCC 43504) was employed.

### 2.3. DNA Extraction and* Helicobacter pylori* 16S rRNA Gene Amplification

Based on the PCR technique, suspected colonies were identified as* H. pylori*. Genomic DNA was extracted from the colonies with typical characters of* H. pylori* using a DNA extraction kit for cells and tissues (Roche Applied Science, Germany, 11814770001) according to the manufacturer's instructions and its density which was assessed by optic densitometry. Extracted DNA was amplified for the 16S rRNA gene (primers: HP-F: 5′-CTGGAGAGACTAAGCCCTCC-3′ and HP-R: 5′-ATTACTGACGCTGATTGTGC-3′) [14]. PCR reactions were performed in a final volume of 50 *µ*L containing 5 *µ*L 10x buffer + MgCl_2_, 2 mM dNTP, 2 unit Taq DNA polymerase, 100 ng genomic DNA as a template, and 25 picomole of each primer. PCR was performed using a thermal cycler (Eppendorf Co., Germany) under the following condition: an initial denaturation for 2 minutes at 94°C; 30 cycles of 95°C for 30 s, 60°C for 30 s, and 72°C for 30 s; and a final extension at 72°C for 8 min.

### 2.4. Genotyping of* vacA* Gene of* Helicobacter pylori*

Presence of the genotypes of* vacA* alleles (*s1a*,* s1b*,* s1c*,* m1a*,* m1b,* and* m2*) was determined by PCR. The primer sequences are shown in Table 1 [15].

The PCR was performed in a total volume of 50 *μ*L containing 1 *μ*M of each primer, 1 *μ*L of genomic DNA (approximately 200 ng), 1 mM of dNTPs mix (invitrogen), 2 mM of Mgcl_2_, and 0.05 U/*μ*L Taq DNA polymerase (invitrogen). PCR amplifications were performed in an automated thermal cycler (Biometra Co., Germany). The following cycle conditions were used for PCR amplification: 32 cycles of 45 s at 95°C, 50 s at 64°C, and 70 s at 72°C. All runs included one negative DNA control consisting of PCR grade water and two or more positive controls (*H. pylori* 26695,* H. pylori* J99,* H. pylori* SS1,* H. pylori* Tx30,* H. pylori* 88-23, and* H. pylori* 84-183).

### 2.5. Gel Electrophoresis

The PCR amplification products (10 *μ*L) were subject to electrophoresis in a 1% agarose gel in 1x TBE buffer at 80 V for 30 min and stained with ethidium bromide, and images were obtained in UVIdoc gel documentation systems (UK). The PCR products were identified by 100 bp DNA size marker (Fermentas, Germany).

### 2.6. Statistical Analysis

Using SPSS 16.0 statistical software (SPSS Inc., Chicago, IL, USA), Chi-square test and Fisher's exact two-tailed test analysis were performed, and differences were considered significant at values of *P* < 0.05. Distributions of genotypes of* H. pylori* isolated from food stuff were statistically analyzed.

## 3. Results and Discussion

All of the milk and meat samples were examined using the culture and PCR techniques.  Table 2 shows the distribution of* H. pylori* in the milk and meat samples. Of 820 meat and milk samples, 197 (24.02%) were positive for* H. pylori*. Of 420 milk and 400 meat samples, 92 (21.90%) and 105 (26.25%) were positive for* H. pylori*, respectively (Table 2).

The most commonly contaminated milk and meat samples were raw sheep milk (29.16%) and raw sheep meat (37%). There were no statistically significant differences among the incidence of bacteria in milk and meat samples. There were statistically significant differences in the incidence of* H. pylori* between sheep and camel milk (*P* = 0.033) and between sheep and camel meat (*P* = 0.048). Distribution of* vacA* genotypes of the* H. pylori* strains of meat and milk samples is shown in Table 3.

**Figure 1:**
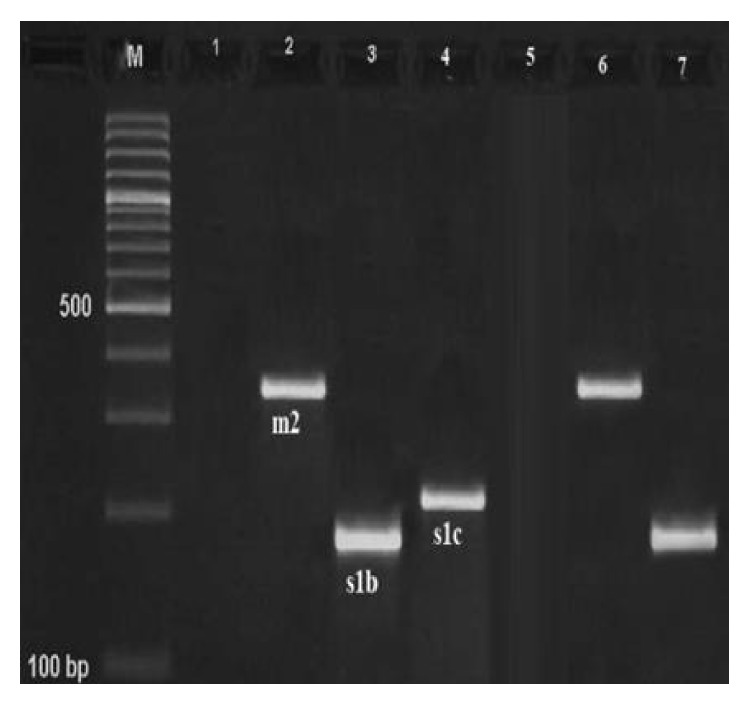
Results of the gel electrophoresis for identification of* m2*,* s1b,* and* s1c* genotypes of the* H. pylori* strains of milk and meat samples. Line 5: negative control, line 7: positive controls, 1: negative sample, M: 100 bp DNA ladder (Fermentas, Germany), and numbers 2–4: positive samples for* m2* (352 bp),* s1b* (187 bp), and* s1c* (213 bp) alleles.

**Figure 2 fig2:**
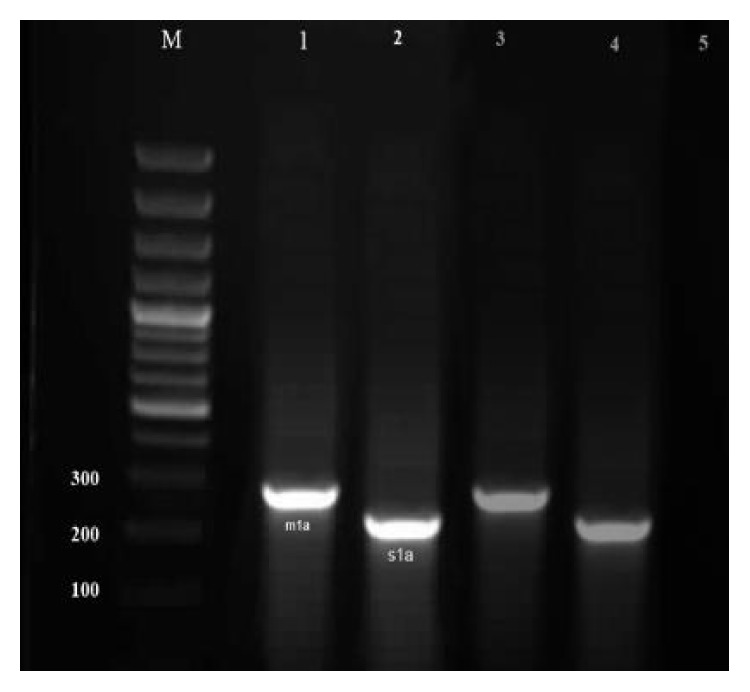
Results of the gel electrophoresis for identification of* m1a* and* s1a* genotypes of the* H. pylori* strains of milk and meat samples. Lanes 3 and 4: positive controls, 5: negative control, M: 100 bp DNA ladder (Fermentas, Germany), and numbers 1-2: positive samples for* m1a* (290 bp) and* s1a* (213 bp) alleles, respectively.

**Figure 3:**
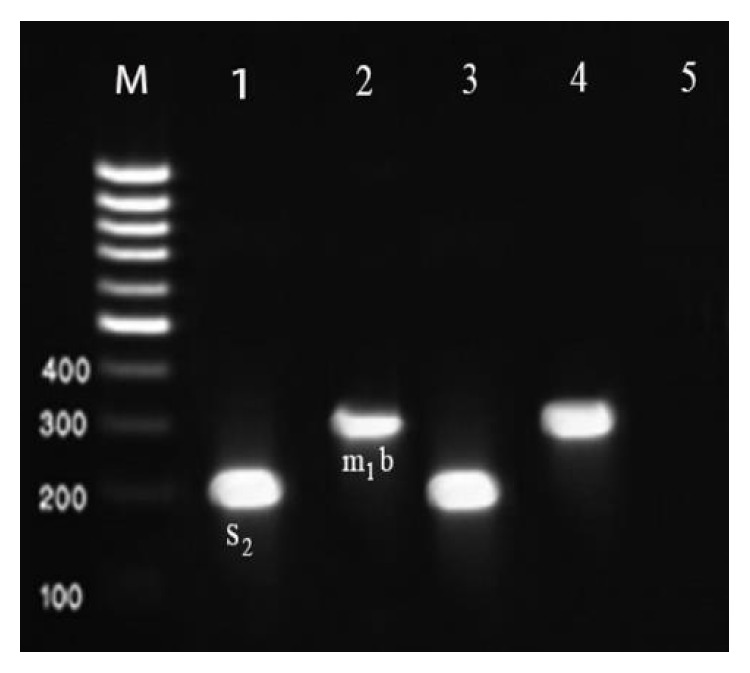
Results of the gel electrophoresis for identification of* m1b* and* s2* genotypes of the* H. pylori* strains of milk and meat samples. Lanes 3 and 4: positive controls, 5: negative control, M: 100 bp DNA ladder (Fermentas, Germany), and numbers 1-2: positive samples for* s2* (199 bp) and* m1b* (291 bp) alleles, respectively.

Results of the gel electrophoresis of PCR products for amplification of various genotypes of* vacA* gene are shown in Figures 1 [Fig fig2]–3.

Significant difference was found between the type of samples and prevalence of genotypes (*P* < 0.05). Fourteen different genotypic combinations are shown in Table 4. The most commonly detected combined genotypes were* m1as1a* (68.52%),* m1as1b* (60.40%),* m1bs1b* (55.83%), and* m1bs1a* (53.29%).

Results of current study showed that raw milk and meat samples were reservoir for* H. pylori*. Total prevalences of* H. pylori* in raw cow, sheep, goat, buffalo, and camel milk samples of our survey were 20.83%, 29.16%, 18.75%, 24%, and 10%, respectively. Rahimi and Kheirabadi [3] reported that the incidence of* H. pylori* in raw cow, sheep, goat, buffalo, and camel milk samples of Iranian herds was 1.41%, 12.20%, 8.70%, 23.4%, and 3.6%, respectively, which was lower than our results. In a study carried out in Italy,* H. pylori* was detected in 50%, 33%, and 25.6% of raw cow, sheep, and goat milk, respectively, which was higher than our results [16]. In a study conducted in Japan,* H. pylori* was detected in 72.2% of raw cow milk samples [17]. Total distribution of* H. pylori* in the milk samples of Greek [18] and American [4] herds was 20% and 60%, respectively. Recent clinical investigation among Iranian cows showed that 16% of milk and 40% of feces samples of seropositive herds were infected with* H. pylori* [2].

Total prevalence of* H. pylori* in cow, sheep, goat, buffalo, and camel meat samples of our survey was 25%, 37%, 22%, 28%, and 14%, respectively, which was entirely contrary to the results of Stevenson et al. [19]. They suggested that transmission of* H. pylori* from beef and beef products is not a primary factor in the high prevalence of this bacterium in humans. Moreover, Mhaskar et al. [20] reported that the prevalence of peptic ulcer and* H. pylori* infection were entirely higher in those patients who have used meat and meat products (Odds Ratio (OR): 2.35, 95% and Confidence Interval (CI): 1.30–4.23) and restaurant foods (OR: 3.77, 95% CI: 1.39–10.23) in their main meals.

The possibility that* H. pylori* may be a zoonosis first rose the publication of two epidemiological studies that exhibited that the prevalence of* H. pylori* infection in abattoir and meat workers was significantly increased in comparison with the subjects that were not involved in handling animals or meat [21, 22]. This hypothesis is further reinforced by the demonstration of* H. pylori* in the gastric mucosa of calves, pigs, and horses and its isolation from sheep's gastric tissue and milk [4], suggesting that these animal species may act as reservoirs and spreaders of* H. pylori*. Findings of Momtaz et al. [1] have conclusively proved the zoonotic aspects of* H. pylori*. They showed that the* vacA s1a/m1a* was prominent* H. pylori* genotype in all cow, sheep, and human beings clinical samples. They showed 3.4–8.4% variability and 92.9–98.5% homology between sheep and human samples [1].

High prevalence of* H. pylori* in the milk (29.16%) and meat (37%) samples of sheep of our study suggested that sheep may be the natural host of* H. pylori*. Dore et al. [4] reported that* H. pylori* DNA was demonstrated in 60% (38/63) of milk samples and in 30% (6/20) of sheep tissue samples. They showed that the* vacA* gene was amplified in five of 38 milk samples, and in two of six sheep tissue samples, respectively. Sequence analysis of 16S rRNA PCR products from* H. pylori* strains [4] investigation demonstrated 99% identity with* H. pylori*.

Genotyping using* vacA* virulence marker gene is considered as one of the best approaches for studying of the correlations between* H*.* pylori* isolates from different samples [1].* H. pylori* strains of milk and meat samples of our study showed similar status in the distribution of* vacA* genotypes. Totally, the most commonly detected genotypes in milk and meat samples were* s1a* (86.80%),* m1a* (79.18%),* s1b* (69.54%), and* m1b* (63.45%). Various genotypes of* vacA* strains were the most commonly detected genotypes in the studies of Linpisarn et al. (Thailand) [23], López-Vidal et al. (Mexico) [24], and Rudi et al. (Germany) [25]. The high presence of* m1as1a* and* m1as1b* genotypes has been reported previously from Iran [13] and Germany [25] but far different results have been reported from Thailand [23] and Mexico [24].

According to the high prevalence of pathogenic strains of* H. pylori* in milk and meat samples especially in those collected from sheep and also based on the considerable consumption of milk and meat in their raw forms in some areas of the world [26–28], consumption of these food products in their raw forms should be stopped. On the other hand, thorough cooking of meat and pasteurization of milk can prevent the presence and also transmission of pathogenic bacteria like* H. pylori*.

## 4. Conclusions

*H. pylori* which is harbored from milk and meat samples are similar in genotype of the* vacA *allele with isolates recovered from human. Also, since there was a high similarity in the genotyping pattern of* H. pylori* DNA among milk and meat samples and human specimens of other investigations, it is suggested that raw milk and meat samples are the sources of the bacteria and that they entered the human population in period of time. On the other hand, diversity of* H. pylori* genotypes between milk and meat samples with the clinical isolation of other studies suggested that consumption of contaminated milk and meat with* H. pylori* strains may be a threat to human health.

## Figures and Tables

**Table 1:** Oligonucleotide primers used for genotyping of *Helicobacter pylori* isolated from foods with animal origin in Iran [15].

*vacA alleles*	Primer sequence (5′-3′)	Size of product (bp)
*s1a*	F: CTCTCGCTTTAGTAGGAGC R: CTGCTTGAATGCGCCAAAC	213

*s1b*	F: AGCGCCATACCGCAAGAG R: CTGCTTGAATGCGCCAAAC	187

*s1c*	F: CTCTCGCTTTAGTGGGGYT R: CTGCTTGAATGCGCCAAAC	213

*s2*	F: GCTAACACGCCAAATGATCC R: CTGCTTGAATGCGCCAAAC	199

*m1a*	F: GGTCAAAATGCGGTCATGG R: CCATTGGTACCTGTAGAAAC	290

*m1b*	F: GGCCCCAATGCAGTCATGGA R: GCTGTTAGTGCCTAAAGAAGCAT	291

*m2*	F: GGAGCCCCAGGAAACATTG R: CATAACTAGCGCCTTGCA	352

**Table 2:** Distribution of* Helicobacter pylori* in various types of raw milk and meat samples.

Types of samples	Number of samples collected	Positive results for *H. pylori* (%)
Cow milk	120	25 (20.83)
Sheep milk	120	35 (29.16)
Goat milk	80	15 (18.75)
Buffalo milk	50	12 (24)
Camel milk	50	5 (10)
Total raw milk	**420**	**92 (21.90)**
Cow meat	100	25 (25)
Sheep meat	100	37 (37)
Goat meat	100	22 (22)
Buffalo meat	50	14 (28)
Camel meat	50	7 (14)
Total raw meat	**400**	**105 (26.25)**
Total	**820**	**197 (24.02)**

**Table 3:** Distribution of *vacA* genotypes in *Helicobacter pylori* strains of meat and milk samples.

Types of samples (number of positive results)	Distribution of *vacA* genotypes (%)						
	*s1a*	*s1b*	*s1c*	*s2*	*m1a*	*m1b*	*m2*
Cow milk (25)	20	18	14	9	19	17	8
Sheep milk (35)	32	23	16	12	27	20	10
Goat milk (15)	12	10	8	5	10	9	4
Buffalo milk (12)	10	8	5	7	8	8	2
Camel milk (5)	5	4	—	2	3	3	—
Total raw milk **(92)**	**79 (85.86)**	**63 (68.47)**	**43 (46.73)**	**35 (38.04)**	**67 (72.82)**	**57 (61.95)**	**24 (26.08)**
Cow meat (25)	22	19	14	10	20	19	9
Sheep meat (37)	34	26	20	13	29	21	13
Goat meat (22)	19	15	11	8	17	16	7
Buffalo meat (14)	11	9	6	9	10	9	5
Camel meat (7)	6	5	1	3	3	3	1
Total raw meat **(105)**	**92 (87.61)**	**74 (70.47)**	**52 (49.52)**	**43 (40.95)**	**89 (84.76)**	**68 (64.76)**	**35 (33.33)**
Total **(197)**	**171 (86.80)**	**137 (69.54)**	**95 (48.22)**	**78 (39.59)**	**156 (79.18)**	**125 (63.45)**	**59 (29.94)**

**Table 4:** Distribution of combined genotypes of *Helicobacter pylori* isolated from Iranian raw milk and meat samples.

Genotypes	Prevalence (%)^*∗*^
*M1as1a*	135 (68.52)
*M1as1b*	119 (60.40)
*M1bs1a*	105 (53.29)
*M1bs1b*	110 (55.83)
*M1as1c*	70 (35.53)
*M1bs1c*	58 (29.44)
*M2s1a*	40 (20.30)
*M2s1b*	31 (15.73)
*M2s1c*	26 (13.19)
*M2s2*	19 (9.64)
*M1as2*	56 (28.42)
*M1bs2*	44 (22.33)

^*∗*^Percent of positive genes from total of 197 positive samples.
